# Metabolomics and microscopic profiling of flaxseed meal- incorporated Peda

**DOI:** 10.1016/j.fochms.2024.100217

**Published:** 2024-08-22

**Authors:** Sachin Maurya, Tarun Verma, Ankur Aggarwal, Manish Kumar Singh, Abhishek Dutt Tripathi, Ankur Trivedi

**Affiliations:** a Department of Dairy Science and Food Technology, Institute of Agricultural Sciences, Banaras Hindu University, Varanasi, Uttar Pradesh, India; b Department of Food Technology, School of Engineering and Technology, Mizoram University, Aizawl, Mizoram, India; c Department of Dairy Technology, National Dairy Research Institute, Karnal, Haryana, India

**Keywords:** Functional food, *Peda*, Flaxseed meal, Metabolomics, Bioactive compounds

## Abstract

Functional dairy foods are in high demand due to their convenience, enhanced nutrition, intriguing flavors, and natural ingredients. The valorization of flaxseed by-products can potentially boost the functionality of these foods. This work involves the optimization of flaxseed meal powder (2%, 2.5%, 3%) during *Peda* preparation based on sensory and textural attributes. The optimized *Peda* (2%) exhibited significantly reduction in moisture (39.6%) and water activity (18.9%), while significantly increasing crude fiber (1.88%), protein (26.4%), fat (8%) and DPPH inhibition (274.5%) as compared to control *Peda*. Scanning electron microscopy of the optimized *Peda* revealed the surface displayed a dense, uneven texture, heavily coated with fat, and intergranular spaces filled with milk serum. Twenty-three primary compounds were recognized in high-resolution mass spectrometry (HR-MS), including 6 organic acids, 6 amino acids, 3 fatty acids, 3 other metabolite derivatives, 2 lipids, 2 bioactive components, and 1 sugar. Besides gas chromatography mass spectrometry (GC–MS) found six separate types of fatty acids. These compounds have been proven to possess various bioactivities, such as promoting brain activity, antioxidant, anti-diabetic, anti-inflammatory, cardiovascular-protective effects, etc. Flaxseed meal, as a plant-based substitute for dairy ingredients, offers a sustainable and healthy alternative, making flaxseed-incorporated *Peda* a functional food.

## Highlights

• Metabolomics of optimized *Peda* was done using two different omics tools.
• Optimization (2% FMP) reveals high sensorial and textural properties.
• Enhanced level of fat, protein and antioxidants were seen in the best formulation.
• HR-MS and GC–MS identified 22 bioactives and six key fatty acids respectively.
• FMP added *Peda* can be functional dairy product with plant-based substitution.

## Graphical abstract

**Figure:**
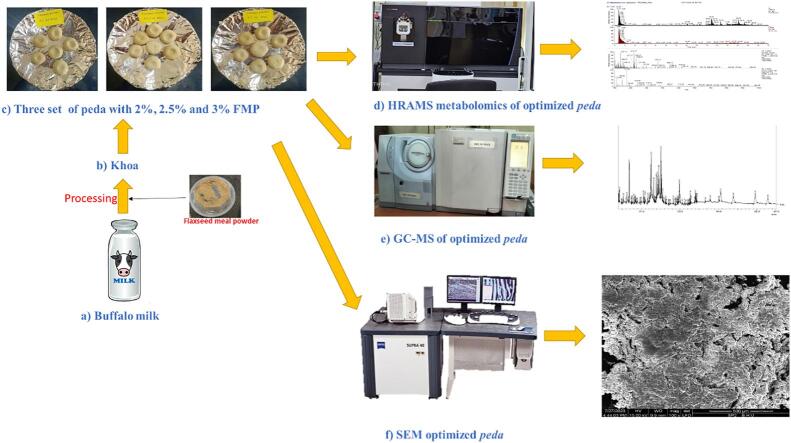

## 1 Introduction

A substantial amount of oilseed plant produce ends up as by-products and waste during processing, annually (Rao et al., 2021). Often, these by-products are either discarded or used as animal feed. Nevertheless, many of these by-products are rich in bioactive compounds that can be utilized to develop innovative food products through different valorization methods (Łopusiewicz et al., 2019).

Increasing consumer awareness of health issues has led to a higher demand for plant-based functional foods (Łopusiewicz et al., 2019). Food companies are increasingly seeking alternatives to address health and environmental challenges through the development of new plant-based functional foods. Transitioning to a fully plant-based diet can be difficult, so a partial substitution approach may benefit both consumers and the industry. Combining dairy and plant ingredients represents a promising alternative, offering potential health benefits and easing the transition to plant-based products. Desiccated dairy products like “*Peda*” could serve as an effective base for incorporating oilseed processing by-products through partial substitution (Ravindra et al., 2022).

*Peda* is a sweetened heat-desiccated traditional dairy product that is widely consumed in the Indian sub-continent, and has its roots in the culture of the region since ancient times (Asgar & Chauhan, 2019). This dairy product has its key base material, known as *Khoa* which is made by heat-concentrating the buffalo milk in an open pan until it achieves a dough-like consistency. Researchers have noted that khoa made from buffalo milk not only exhibits increased yield but also elucidates superior sensory quality and enhanced textural attributes (Choudhary et al., 2017).

Flaxseed (*Linum usitatissimum L.*) is an annual plant within the *Linaceae* family that has gathered attention from nutritionists and food scientists because of its immense potential for utilization in the functional food product development that carries active components like α-Linolenic acid, lignin, and dietary fiber (Kajla et al., 2023). With increasing consumer demand for functional foods that could offer enhanced health advantages, flaxseed has emerged as a promising ingredient that is gaining popularity in diets tailored to specific health objectives (Gul et al., 2016). The flaxseed oil processing industry generates a valuable by-product i.e., flaxseed meal (FM) which contains ∼ 40 % protein and is presently utilized for protein supplements, fertilizers, and feed development (Wu et al., 2019). The valorization of FM in the dairy product matrix could be the sustainable and nutritional alternative for the use of this by-product in functional food development.

Untargeted metabolomics is always an intriguing tool for analyzing the comprehensive primary and secondary metabolites in a product and was employed for metabolic activity analysis of these functional foods. The application of omics technology, particularly the high-resolution mass spectroscopy (HR-MS) approach is improving the detection of bioactive and putative markers in the valorized dairy-based products (Jaspal et al. (2024). Gas chromatography-mass spectroscopy (GC–MS) is also an effective and efficient tool for detailed volatile compound profiling of the food product. The application of such robust methodologies in the characterization of bioactive components of a valorized Indian dairy product could reveal the overall functionality of the product.

In the present study, flaxseed meal powder (FMP) was added to the base matrix of *Peda* in varying concentrations to develop functional dairy products. Furthermore, a specialized bioactive metabolite search based on high-resolution mass spectroscopy (HR-MS) and gas chromatography-mass spectrometry (GC–MS) were performed on FMP-incorporated *Peda* to highlight the functionality of the product.

## 2 Materials and methods

### 2.1 Reagents and materials

Mature whole flaxseed (10 kg) was sourced from the agricultural unit of the Banaras Hindu University, Varanasi followed by manually cleaned to remove impurities, cold-pressed to obtain oil defatted meal (Lee et al., 2023). The FM was broken and other ingredients were individually roasted with a small amount of oil in a frying pan for 5 min at 80 °C to remove any off-flavor. After cooling the meal was ground separately in a laboratory mixer (Kenstar, Mumbai) at room temperature to obtain FPM. Formic acid (FA) and methanol (MeOH) were both of LC–MS grade and sourced from Sigma Aldrich (Bengaluru, India). Additional materials such as syringes, polytetrafluoroethylene (PTEF) filters (25  mm × 0.22  µm), amber vials (2 ml), polypropylene falcon tubes (50  ml), test tubes, glass beakers, etc. were purchased from the Hi-Media (Mumbai, India).

### 2.2 Development of flaxseed meal powder added *Peda*

**Fig. 1:**
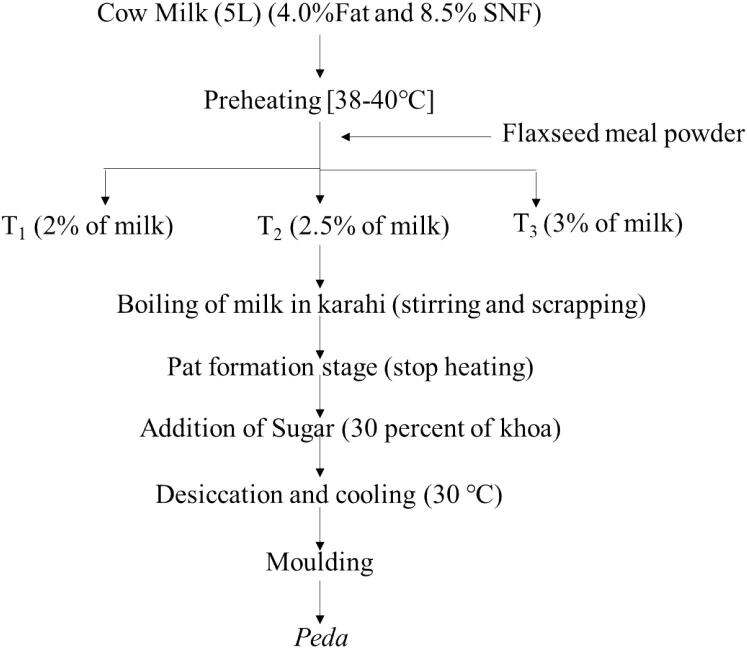
The process flow diagram of flaxseed meal powder incorporated *Peda* making.

During the development of *Peda*, 5 L of milk (6.0 % fat, 9.0 % SNF) were sourced from the Dairy Farm, Banaras Hindu University. Following the methodology outlined by Badola et al. (2023) with slight alterations, four-set *Peda* treatments T_1_: (milk + 2 % FMP), T_2_: (milk + 2.5 % FMP), T_3_: (milk + 3 % FMP) and control T_0_: (milk only) were formulated, each replicated three times. The meal powder was limited to 3 % to maintain dough consistency (Fig. 1). The process involved heating the milk to ∼ 80 °C with continuous stirring, then cooling until the desired consistency was reached. The *Peda* was optimized using sensorial analysis (20 semi-trained panellists) and textural attributes (Model: CT3, Brookfield, Mumbai, India). The sample reveals formulation having 2 % FMP has high sensorial and textural properties.

### 2.3 Physico-chemical analysis

In the T_o_ and T_1_ samples fat, moisture, protein, total carbohydrate, fiber, antioxidants (mg GAE), and ash content were determined using the method described by Pratap et al., 2019, while reducing and non-reducing sugars were analysed (Roy et al., 2024). Water activity (a_w_) (Rotronic AG, Switzerland) of control and optimized *Peda* samples were measured at 25 °C (Hirpara et al., 2015).

### 2.4 Microscopic examination of *Peda* structure

Scanning electron microscope (SEM) was utilized to check the morphological characteristics of both Peda samples that were controlled and those that had been incorporated with FMP. This analysis was conducted using a Zeiss SUPRA-40 model located in India’s Mumbai city and took place at 100 × magnification.

### 2.5 HR-MS metabolomics of *Peda*

Untargeted high-resolution mass spectrometry using Thermo Fisher equipment was conducted following the procedure outlined by Jaspal et al. (2024) with slight modification. For HR-MS sample preparation, 1.5 ml of an 80:20 methanol mixture was added to 100 mg of the optimized samples. The mixture was homogenized at a speed of 750 rpm for 30 min at 25 °C. It was then centrifuged at 3500 rpm for 10 min, also at 25 °C. The resulting supernatant was filtered using a PTFE syringe filter with 0.22 μm pores. For this purpose, 4 μl of the filtered sample were introduced into a Hypersil GOLD™ C18 RP-HPLC column holding particles of dimensions 1.9 μm in size, 2.1 mm in diameter and 100 mm in length. This resulted to chromatographic separation attained in a gradient system that varied from 5 % MeOH to 90 % MeOH within 30 min while flowing at 300 μL/min meanwhile maintaining 40 °C within the column. The Thermo Scientific Orbitrap Eclipse equipped with Ultra High Performance Liquid Chromatography UHPLC, and heated electrospray ionization (HESI) source were employed to conduct three measurements of the optimized Peda (T1). Data dependent analysis (DDA) was carried out using Compound Discoverer 3.3.2.31 and different online databases. The analysis was performed by means of a resolution of 15,000 under positive (3500 V) and negative (2500 V) ionization modes. With this approach, one could identify, identification, and grouping of unknown compounds, estimate chemical formulas, find similar spectra among their spectral similarity searches and what not; consequently, these findings could be utilized in creating molecular groups through corresponding connections within the molecular networks made out from them.

### 2.6 Analysis using gas chromatography-mass spectrometry (GC–MS)

We analyzed quantitatively the methanolic crude extract from the optimized Peda through a Shimadzu QP 2010 PLUS GC–MS system which was employed with a 30 m × 0.25 mm × 0.25 µm film thickness capillary column (Hossain & Rahman 2011). The injection was splitless with 1-minute purge time. The flow rate of helium carrier gas was 1 ml/min. After being set at 50 °C for 3 mins, the column temperature was raised to 80 °C at a pace of 5 °C/min, and then to 340 °C at a pace of 10 °C/min. The inlet temperature was 250 °C, detector temperature was 340 °C, and a 4 mins solvent delay was applied. In peak identification, mass spectra is matched against NIST 08 and NIST 08 s library and also compared with published data.

### 2.7 Statistical analysis

SPSS for Windows release 9.0 (SPSS.16, Chicago, USA) was employed to conduct the ANOVA tests. The means were compared using data from the least significant difference multiple range analysis. To assess variations between treatments multiple-range test was utilized. Statistical significance was evaluated at the 5 % level and each experiment was replicated three times (*n = 3*).

## 3 Results and discussion

The current study focuses on creating Peda enriched with FMP to enhance its functional properties and its foodomics metabolite profiling was done.

### 3.1 Proximate composition, sensory and textural attributes of *Peda*

Different concentrations of FMP were incorporated into the development of functional Peda for optimization purposes using sensory and textural attributes. The sample of Peda containing 2 % FMP (T1) treatment was the best optimized in terms of sensorial and textural attributes in present study. FMP-*Peda* when compared to the control (T_0_), has a higher fat content mainly due to FMP in a diet may contribute to increased healthy fat intake (Zhang et al., 2023). It also exhibits a significant increase in protein and fiber, making it a valuable plant-based protein source with digestive and potential weight management benefits (Xie et al., 2023). The organoleptic characterization of FMP *Peda* were acceptable when flaxseed powder was added to yoghurt as reported by Marand, et al., 2020. The addition of flaxseed meal powder significantly increased (P<0.05) the hardness of *Peda*, likely due to the high total solid content in the flaxseed meal powder. Suresh and Jha (1994) observed that increased total solids in milk were strongly correlated with greater hardness in khoa. Furthermore, adding flaxseed meal powder to *Peda* significantly (P<0.05) decrease adhesiveness. Bhat et al. (2018) also reported that flaxseed altered internal bonds in yogurt, reducing adhesiveness and enhancing cohesiveness. The optimization results of the physicochemical, sensory and textural attributes of FMP-*Peda* with control *Peda* are shown in Table 1.

**Table 1:** Physicochemical sensory and textural attributes of the control and FMP incorporated *Peda.*

**Treatment**	**Physico-Chemical Analysis**	
	**Control *Peda***	**FMP-*Peda***
**Parameter (percent)**	**T_0_**	**T_1_**
**Fat**	30.14 ± 0.21^b^	32.55 ± 0.08^a^
**Moisture**	19.56 ± 0.22^a^	11.82 ± 0.31^b^
**Protein**	15.93 ± 0.13^a^	20.13 ± 0.11^b^
**Fibre**	0.00^a^	1.88 ± 0.31^b^
**Ash**	2.82 ± 0.01^a^	2.80 ± 0.03^a^
**Reducing sugar**	31.43 ± 1.10^a^	30.11 ± 1.01^a^
**Non-Reducing Sugar**	0.49 ± 0.01^a^	0.54 ± 0.01^a^
**Total Solid**	80.43 ± 1.31^a^	87.03 ± 1.21^b^
**Antioxidant (mg/GAE)**	9.4 ± 0.31^a^	35.2 ± 2.11^b^
**Water Activity**	0.8070 ± 0.01^a^	0.6548 ± 0.01^b^

Sensory analysis		
**Colour and Appearance**	8.6 ± 0.31^a^	8.3 ± 0.17^a^
**Body and Texture**	8.4 ± 0.34^a^	8.2 ± 0.15^a^
**Flavour and taste**	8.5 ± 0.28^a^	8.2 ± 0.20^a^
**Overall Acceptability**	8.48 ± 0.27^a^	8.3 ± 0.21^a^

Texture analyser		
**Hardness (g)**	1516.87 ± 97.2^b^	1982.89 ± 148.32^a^
**Adhesiveness (g sec)**	−13.66 ± 1.36^b^	−24.37 ± 2.13^a^
**Springiness**	0.293 ± 0.02^a^	0.312 ± 0.05^a^
**Cohesiveness**	0.123 ± 0.006^a^	0.144 ± 0.005^a^

Note: The values are presented as mean ± standard deviation, based on three samples (n = 3). Significant differences at the p ≤ 0.05 level are indicated by different superscripts (a,b) within each column.

### 3.2 Micro-structure of the *Peda*

**Fig. 2:**
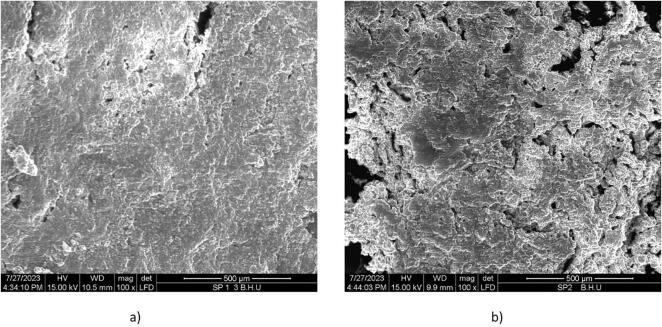
Illustration of the scanning electron micrographs (a) control *Peda* sample and (b) optimized *Peda* sample.

The microstructure analysis with SEM reveals that extended heat treatment in control *Peda* (Fig. 2a) caused the formation of complexes between casein and whey proteins that have undergone amalgamation with lactose and add sugar leading to thick protein bridges. Similar finding was also reported by Mehta, 2018. Commercially available plain *Peda* (control sample) showed large clusters of lactose crystals and increased void space may be due to non-uniform kneading processes during production (Das et al., 2018). In optimized sample T_1_ (Fig. 2b), the surface displayed a dense and uneven texture, heavily coated with fat with intergranular spaces filled with milk serum, mucilages, and fat mainly due to addition of FMP in *Peda* sample.

### 3.3 HR-MS and GC–MS analysis of optimized *Peda*

**Fig. 3:**
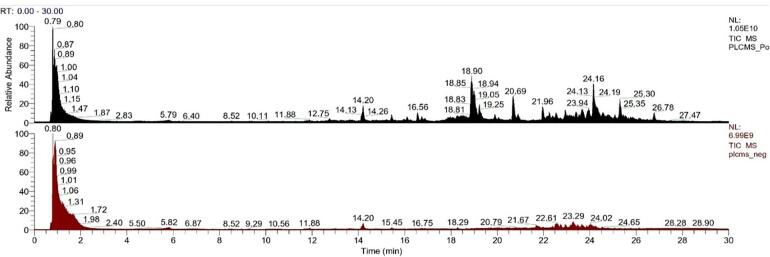
The comprehensive mass spectrum representing all the metabolites identified through HR-MS in the optimized Peda sample.

The metabolites profile study of optimized *Peda* represents an effort to perform liquid chromatography coupled with mass spectrometry-based omics for the bioactive compounds analysis in the optimized sample having a 2 % concentration of FMP as its primary constituent. We employed an untargeted approach utilizing an orbitrap mass spectrometer working in DDA acquisition mode with an electrospray ionization (ESI) process in both negative and positive modes. The resulting representative metabolome mass spectrum (Fig. 3) of this functional product revealed a distinctive variation pattern, suggesting differential alignment of metabolites in the various concentrations.

After completing the mass spectrometry omics analysis for the FMP- *Peda*, the results disclosed a sum of 1654 differential metabolites, formed from various interactions within the *Peda* matrix constituents. The representative mass spectrum shows 37 annotated peaks highlighting major metabolites of primary and secondary classifications, out of which 23 important bioactive metabolites are listed in Table 2. Six of which are organic acids, another six are amino acids, three are fatty acids, three are other derivatives of metabolites, two are lipids, two are bioactive substances, while one is a sugar. All these exhibiting diverse physiological functions in the human body including antioxidative, antimicrobial, antidiabetic, anti-inflammatory, anti-cardiovascular, brain activity, neurogenerative, and lipid metabolism roles. The list in Table 2 describes important metabolomic compounds accompanied by their detailed chemical formulas, molecular weights, retention times (RT), total areas covered by the mass spectral peaks and respective functions.

**Table 2:** The major metabolites obtained from the HR-MS metabolomics analysis of the optimized *Peda*.

**Name**	**Formula**	**Annot. Delta Mass [ppm]**	**Calc.** **MW**	***m*/*z***	**RT** **[min]**	**Area** **(Max)**	**Compound property**	**References**
Fatty Acids and Derivatives								
Ethyl eicosapentaenoic acid	C_22_ H_34_ O_2_	−1.62	330.2554	329.248	24.755	2,222,550	Anti-CVD	Saberi et al., 2023
Hexadecanamide	C_16_ H_33_ N O	−0.48	255.2561	256.2634	23.325	916,664,519	Anti-inflammatory, analgesic	Mazzari et al., 1996
12-methyltridecanoate	C_17_ H_34_ O_4_	0.25	302.2458	285.2425	22.578	462230005.5	Lipid metabolism	Zhang et al., 2019

Sugars								
Trehalose	C_12_ H_22_ O_11_	−2.1	342.1155	341.1082	1.276	14,122,600,563	Antioxidant	Chen et al., 2022

Amino Acids and Derivatives								
D-Pyroglutamic Acid	C_5_ H_7_ N O_3_	1.2	129.0428	130.0501	0.895	463750859.4	Anti-fungal	Ai et al., 2022
Creatine	C_4_ H_9_ N_3_ O_2_	1.59	131.0697	132.077	0.876	6,919,735,256	Anti-inflammatory	Cordingley et al., 2022
Acetyl-L-carnitine	C_9_ H_17_ N O_4_	0.74	203.1159	204.1232	0.882	3,568,630,660	Brain activity	Ferreira and McKenna, 2017
L-Glutamic acid	C_5_ H_9_ N O_4_	1.16	147.0533	148.0606	0.895	1,226,252,520	Brain activity	Coyle et al., 2002
1-Stearoylglycerol	C_21_ H_42_ O_4_	0.56	358.3085	341.3052	24.47	375541062.3	Lipid metabolism	Mondul et al., 2014
1-Oleoyl-2-hydroxy −sn-glycero-3-PE	C_23_ H_46_ N O_7_ P	−1.12	479.3007	478.2934	23.864	332356025.7	Brain activity	Castilla-Ortega et al., 2014

Organic Acids								
2-Furoic acid	C_5_ H_4_ O_3_	−2.26	112.0158	111.0085	0.918	1,662,984,851	Antioxidant	Zahirović et al., 2023
Acetyl-β-methylcholine	C_8_ H_17_ N O_2_	1.08	159.1261	160.1334	0.896	464884416.7	Brain activity	Uutela et al., 2005
Trans-Aconitic acid	C_6_ H_6_ O_6_	−0.37	174.0164	173.0091	0.877	440591315.2	Antioxidant	Piang-Siong et al., 2017
Indole-3-carboxilic acid	C_9_ H_7_ N O_5_	−1.05	241.0042	239.997	6.873	404,777,580	Anti-inflammatory	Solanki et al., 2020
Hippuric acid	C_9_ H_9_ N O_3_	0.51	179.0583	180.0656	5.195	436241230.9	Anti-bacterial	De Simone et al., 2021
Orotic acid	C_5_ H_4_ N_2_ O_4_	−0.51	156.017	155.0098	0.918	336798390.2	Antioxidant	Hassani et al., 2019

Lipids								
Sphinganine	C_18_ H_39_ N O_2_	0.82	301.2983	302.3056	17.008	10,544 184,010	Anti-aging	Wang et al., 2024
1-palmitoyl-2-oleoyl-*sn*-glycero-3 −phosphocholine	C_42_ H_82_ N O_8_ P	0.64	759.5783	760.5856	24.252	386748300.8	Brain activity	Fogarty et al., 2015

Bioactive Components								
Methyl picolinate	C_7_ H_7_ N O_2_	1.17	137.0478	138.0551	1.092	846,701,305	Antidiabetic	Sanna et al.,2014
Palmitoyl Serinol	C_19_ H_39_ N O_3_	0.62	329.2932	330.3005	19.293	502,451,199	Anti-inflammatory	Seo et al., 2022

Other Metabolites								
Betaine	C_5_*H*_11_ N O_2_	1.33	117.0791	118.0864	1.009	3,048,259,345	Anti-inflammatory	Zhao et al., 2018
Adenosine	C_10_ H_13_ N_5_ O_4_	0.59	267.0969	268.1042	2.783	334064899.8	Brain activity	Boison, 2009
D-Pyroglutamic Acid	C_5_ H_7_ N O_3_	1.2	129.0428	130.0501	0.895	463750859.4	Anti-fungal	Ai et al., 2022

RT: Retention Time: CVD: Cardiovascular disease; Calc. MW: Calculated molecular weight; *m*/*z*: mass/charge.

This prior work (Zhao et al., 2019) publish study focus on flaxseed metabolomics where they identified physiologically significant metabolites including alkaloids and polyphenol compounds. Ethyl eicosapentaenoic acid which are important fatty acid was discovered by research on stirred yogurt manufactured using co‐microencapsulated flaxseed oil as a bioactive ingredient (Saberi et al., 2023). Among the observed major metabolites, few phospholipid derivatives such as 1-oleoyl-2-hydroxy-*sn*-glycero-3-PE and 1-palmitoyl-2-oleoyl-*sn*-glycero-3-phosphocholine are an essential component of cell membranes which can affect neuronal signaling and communication (Castilla-Ortega et al., 2014, Fogarty et al., 2015). Other metabolites also present such as adenosine, acetyl-β-methyl choline, acetyl-L-carnitine, and L-glutamic acid, which regulate neurotransmitter release and neuronal activity (Boison, 2009, Uutela et al., 2005, Ferreira and McKenna, 2017, Coyle et al., 2002). Hippuric acid (possible detection method for COVID-19) is shown in the product due to the conjugation of benzoic acid, glycine and has antibacterial properties (De Simone et al., 2021). Presence of acetyl-L-carnitine in the optimized sample was found which is known as a biomarker of cyanocobalamin deficiency. The metabolite present in sample like orotic acid (Hassani et al., 2019), *trans*-aconitic acid (Piang-Siong et al., 2017), 2-furoic acid (Zahirović et al., 2023), trehalose (Chen et al., 2022) exert their antioxidant effects through various mechanisms including scavenging free radicals, enhancing antioxidant enzyme activity and reducing oxidative stress. As a result, they play a crucial role in protecting cells and tissues from damage induced by reactive oxygen species (ROS).

Presence of some bioactive compounds also found which have including indole-3-carboxylic acid (Solanki et al., 2020), palmitoyl serinol (Seo et al., 2022), hexadecanamide (Mazzari et al., 1996), betaine (Zhao et al., 2018) and creatine (Cordingley et al., 2022) ameliorates the action of controlling the inflammatory discomfort.

The FMP-incorporated Peda contains few metabolites present in its metabolome such as D-Pyroglutamic acid which have antifungal activity potential (Ai et al., 2022), that implies this developed *Peda* is attributing the overall functionality. Additionally. Sphinganine, which is a bioactive compound, is reported to have properties with anti-aging effects (Wang et al., 2024).

The compounds such as 1-stearoyl glycerol, 1–2-methyl tri decanoate, and betaine participate or regulate in lipid metabolism by serving as substrates or intermediates in lipid synthesis or breakdown pathways (Mondul et al., 2014, Yang et al., 2022, Zhang et al., 2019).

**Fig. 4:**
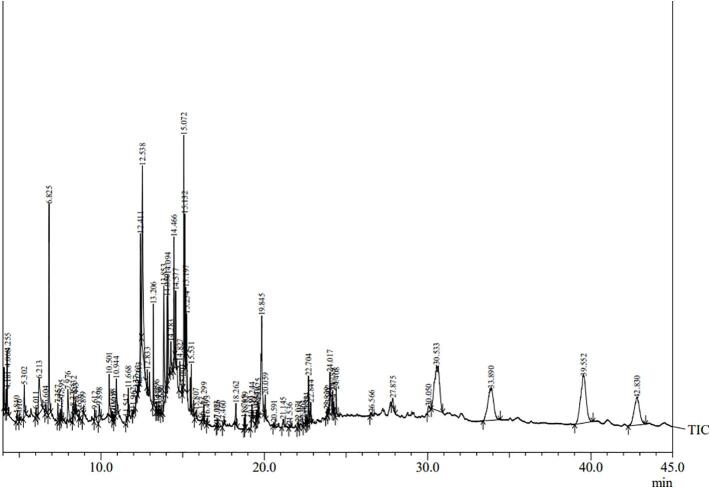
The obtained mass spectrum of the qualitatively fatty acid identification during GC–MS analysis of the optimized *Peda.*

During the GC–MS-equipped metabolomics of the FMP-incorporated *Peda* (Fig. 4), the alignment revealed 92 annotated peaks by the end of the database search (Supplementary Table 1).

Notably, six significant key fatty acids were identified, showing homology with the NIST library, as listed in Table 3. The application of GC–MS also revealed the presence of key bioactive compounds with broad-spectrum functionality, such as methyl alpha-D-glucopyranoside, which showed a high area percentage in the functional dairy product. This compound may result from the breakdown of carbohydrates present in FM (Mani et al., 2011) contributing to the overall sweetness of the product.

**Table 3:** Major Fatty Acids Identified by GC–MS in Optimized Peda Incorporated with Flaxseed Meal Powder.

**Peak**	**RT**	**Area**	**Area%**	**Name**
22	10.501	1,859,413	1.08	3,6,9,13,16-Pentaoxa-2,17-disilaoctadecane (tetracosanoic polyunsaturated fatty acid)
33	12.538	12,595,757	7.29	Methyl alpha-D-Glucopyranoside
60	18.262	842,450	0.49	Palmitic Acid
68	18.829	318,010	4.47	9-Octadecenoic acid, (Linoleic acid)
69	20.059	898,773	0.52	Stearic acid
89	30.533	15,138,537	8.76	Methyl 2-hydroxytetracosanoate (hydroxylated fatty acid)

In the optimized *Peda*, fatty acids such as palmitic acid (0.49 %), and stearic acid (0.52 %) exhibit relatively low area percentages, suggesting a moderate presence of these saturated acids compared to the control *Peda*. Additionally, 9-octadecenoic acid (4.47 %) and methyl 2-hydroxytetracosanoate (8.76 %) exhibit relatively high area percentages, indicating potential benefits for cardiovascular health (Zhang et al., 2023) from the FMP-added *Peda*. Specifically, the presence of 9-octadecenoic acid, a monounsaturated fat, is known for its positive impact on heart health.

Moreover, the identification of 3,6,9,13,16-Pentaoxa-2,17-disilaoctadecane, a tetracosanoic polyunsaturated fatty acid, although at a lower percentage (1.08 %), suggests its role in the nutritional enhancement of the Peda. The broad range of these fatty acids and their varying percentages highlight the nutritional richness and potential health benefits of the optimized Peda.

This work confirms the great importance of food chemistry and the other applied sciences due to their various applications in different fields as shown in a lot of papers published before (Al-Kamali et al., 2014, El Bakri et al., 2023, Mohamed et al., 2022).

## 4 Conclusion

This innovative approach holds substantial potential for creating a functional dairy product with a plant-based source. The incorporation of flaxseed meal powder in the development of *Peda* has resulted in a significant difference related to the physico-chemical and characteristics of the FMP-added *Peda*. The optimized *Peda* exhibited significantly lower levels moisture and water activity but at the same time higher fat, protein, dietary fibre, total solid and antioxidant levels as compared to the control *Peda.* This study also identified major bioactive compounds and fatty acid identification using HR-MS and GC–MS in the optimized *Peda*. The presence of 23 bioactive compounds having brain activity promotion, antioxidant, anti-diabetic, anti-inflammatory, cardiovascular-protective effects, etc. were the major metabolites in the HR-MS probe. The identification of 9-octadecenoic acid by GC–MS (linoleic acid) suggests potential cardiovascular health benefits in the optimized *Peda*. The optimized FMP-incorporated *Peda* can be a functional dairy food highlighted the nutritional richness and potential health benefits of the optimized Peda.

## Funding

No funding was received.

## CRediT authorship contribution statement

**Sachin Maurya:** Writing – original draft, Validation, Investigation, Formal analysis, Conceptualization. **Tarun Verma:** Validation, Supervision, Project administration, Methodology. **Ankur Aggarwal:** Writing – review & editing. **Manish Kumar Singh:** Writing – review & editing. **Abhishek Dutt Tripathi:** Writing – review & editing. **Ankur Trivedi:** Writing – review & editing.

## Declaration of competing interest

The authors declare that they have no known competing financial interests or personal relationships that could have appeared to influence the work reported in this paper.

## Data Availability

Data will be made available on request.
