# Supplementary material for: Metabolomics and microscopic profiling of flaxseed meal- incorporated Peda
Source: Food Chem (Oxf). 2024 Aug 22;9:100217. doi: 10.1016/j.fochms.2024.100217 (PMC11416507; doi:10.1016/j.fochms.2024.100217)
Supplement: Supplementary Data 1 [file mmc1.docx]

**Supplementary Table 1: The 92 annotated peaks from the GC-MS of the developed *peda* incorporated with flaxseed powder.**

| **Peak#** | **R.Time** | **Area** | **Area%** | **Name** |
| --- | --- | --- | --- | --- |
| 1 | 4.068 | 1178235 | 0.68 | CYCLOHEXANE, METHOXY- |
| 2 | 4.181 | 565751 | 0.33 | 2-CYCLOHEXEN-1-OL |
| 3 | 4.255 | 1791050 | 1.04 | 2-Cyclopenten-1-one, 2-hydroxy- |
| 4 | 4.84 | 291905 | 0.17 | 3-Hydroxy-3-methylvaleric acid |
| 5 | 5.01 | 73386 | 0.04 | 2,4-Dihydroxy-2,5-dimethyl-3(2H)-furan-3-one |
| 6 | 5.302 | 1535776 | 0.89 | 2-Hydroxy-gamma-butyrolactone |
| 7 | 6.011 | 268954 | 0.16 | Guanazine |
| 8 | 6.213 | 4325199 | 2.5 | 2-Methylbutanoic anhydride |
| 9 | 6.604 | 428626 | 0.25 | Oxazolidin-2-one, 3-(2-aminoethyl)-4-hydroxy-4-methyl-5 |
| 10 | 6.825 | 7854122 | 4.54 | CYCLOPROPYLMETHANOL |
| 11 | 7.357 | 792403 | 0.46 | 2-Methyl-5-octyn-4-ol, TMS derivative |
| 12 | 7.505 | 400775 | 0.23 | 2-ACETYL-2-HYDROXY-.GAMMA.-BUTYROLACTON |
| 13 | 7.595 | 787584 | 0.46 | 4H-Pyran-4-one, 2,3-dihydro-3,5-dihydroxy-6-methyl- |
| 14 | 7.976 | 2029286 | 1.17 | 2(3H)-Furanone, dihydro-4-hydroxy- |
| 15 | 8.275 | 312272 | 0.18 | Catechol, TMS derivative |
| 16 | 8.332 | 878944 | 0.51 | Phloroglucinol, trimethylsilyl ether |
| 17 | 8.443 | 361168 | 0.21 | 3-Methyl-2-ketobutyric acid tbdms |
| 18 | 8.656 | 144914 | 0.08 | 1,4:3,6-Dianhydro-.alpha.-d-glucopyranose |
| 19 | 8.899 | 327162 | 0.19 | (2,2-Dimethyl-5-[2-(2-trimethylsilylethoxymethoxy)propyl |
| 20 | 9.612 | 621068 | 0.36 | cyclobutanecarboxylic acid, trimethylsilyl ester |
| 21 | 9.898 | 1141006 | 0.66 | TRIMETHYLSILYL ESTER OF 3-METHYL-FURAN-2- |
| 22 | 10.501 | 1859413 | 1.08 | 3,6,9,13,16-Pentaoxa-2,17-disilaoctadecane (tetracosanoic polyunsaturated fatty acid) |
| 23 | 10.698 | 296068 | 0.17 | .alpha.-Ketoisovaleric acid, TMS derivative |
| 24 | 10.81 | 151990 | 0.09 | Methyl 2,2-dimethyl-3,6,9-trioxa-2-silaundecan-11-oate |
| 25 | 10.944 | 2003781 | 1.16 | 2-Hexanol, TMS derivative |
| 26 | 11.547 | 251197 | 0.15 | 4,6-Dioxoheptanoic acid, tris-(O-trimethylsilyl)- |
| 27 | 11.668 | 1251817 | 0.72 | cyclobutanecarboxylic acid, trimethylsilyl ester |
| 28 | 11.962 | 150166 | 0.09 | 2-Hexen-1-ol, (Z)-, TMS derivative |
| 29 | 12.137 | 339416 | 0.2 | Allyl(2-butoxy)dimethylsilane |
| 30 | 12.263 | 126065 | 0.07 | Glycolic acid, acetate, TMS |
| 31 | 12.313 | 87596 | 0.05 | Silane, dimethyl(3-methylbut-3-enyloxy)propoxy- |
| 32 | 12.411 | 5560176 | 3.22 | 2-Penten-1-ol, (Z)-, TMS derivative |
| 33 | 12.538 | 12595757 | 7.29 | Methyl alpha-D-Glucopyranoside |
| 34 | 12.833 | 644237 | 0.37 | SILANE, [1,4-PHENYLENEBIS(OXY)]BIS[TRIMETHY |
| 35 | 13.206 | 2863242 | 1.66 | 2-Ethyl-1,3-bis(trimethylsilyloxy)propane |
| 36 | 13.396 | 311806 | 0.18 | ARABINONIC ACID, 2,3,4-TRIS-O-(TRIMETHYLSILY |
| 37 | 13.477 | 174245 | 0.1 | 2-Oxiranemethanol, .alpha.,alpha.-dimethyl-3-[1-(t-butyldi |
| 38 | 13.556 | 79161 | 0.05 | 4-Mercaptophenol, 2TMS derivative |
| 39 | 13.68 | 193484 | 0.11 | ERYTHRO-PENTITOL, 2-DEOXY-1,3,4,5-TETRAKIS-O |
| 40 | 13.853 | 6643018 | 3.84 | Hexanoic acid, TMS derivative |
| 41 | 14.05 | 4796420 | 2.78 | (2,4-DIFLUORO-BENZYL)-[2-(5-METHOXY-2-METHY |
| 42 | 14.094 | 4997884 | 2.89 | 2,2,18,18-TETRAMETHYL-3,6,10,13,17-PENTAOXA-2, |
| 43 | 14.283 | 1309491 | 0.76 | 2,2,14,14-TETRAMETHYL-3,6,10,13-TETRAOXA-2,14- |
| 44 | 14.466 | 3993101 | 2.31 | D-(-)-Erythrose, tris(trimethylsilyl) ether, ethyloxime (isom |
| 45 | 14.577 | 2304616 | 1.33 | 2-Butene-1,4-diol, (E)-, 2TMS derivative |
| 46 | 14.827 | 2012584 | 1.16 | Hexanoic acid, TMS derivative |
| 47 | 15.003 | 118207 | 0.07 | 3-Deoxy-d-mannoic lactone |
| 48 | 15.072 | 6585535 | 3.81 | D-ERYTHRO-PENTOPYRANOSE, 2-DEOXY-TRIS-O-(T |
| 49 | 15.132 | 3378981 | 1.95 | 2-Methyl-1,4-bis(trimethylsiloxy)butane |
| 50 | 15.197 | 2048837 | 1.19 | 2-Deoxypentofuranose, 3TMS derivative |
| 51 | 15.254 | 1117888 | 0.65 | 2,2,5,8,8-PENTAMETHYL-5-[(TRIMETHYLSILYL)OXY |
| 52 | 15.531 | 1079479 | 0.62 | D-Ribo-Hexonic acid, 3-deoxy-2,5,6-tris-O-(trimethylsilyl) |
| 53 | 15.807 | 586928 | 0.34 | Pyrrolo[1,2-a]pyrazine-1,4-dione, hexahydro- |
| 54 | 16.167 | 6132 | 0 | Pyrrolo[1,2-a]pyrazine-1,4-dione, hexahydro-3-(2-methylpr |
| 55 | 16.299 | 464438 | 0.27 | Myristic acid, TMS derivative |
| 56 | 16.493 | 199115 | 0.12 | Pyrrolo[1,2-a]pyrazine-1,4-dione, hexahydro-3-(2-methylpr |
| 57 | 17.083 | 75866 | 0.04 | 1,2-Pyridazine-3,6-dicarboxylic acid, 4-hexyl-, dimethyl es |
| 58 | 17.126 | 92241 | 0.05 | HEXADECANOIC ACID, METHYL ESTER |
| 59 | 17.46 | 144807 | 0.08 | Pyrrolo[1,2-a]pyrazine-1,4-dione, hexahydro-3-(2-methylpr |
| 60 | 18.262 | 842450 | 0.49 | Palmitic Acid, TMS derivative |
| 61 | 18.769 | 264912 | 0.15 | trans,trans-9,12-Octadecadienoic acid, propyl ester |
| 62 | 18.829 | 318010 | 0.18 | 9-Octadecenoic acid, methyl ester, (E)- |
| 63 | 19.193 | 137783 | 0.08 | 9,12-OCTADECADIENOIC ACID (Z,Z)- |
| 64 | 19.244 | 562727 | 0.33 | cis-9-Hexadecenal |
| 65 | 19.481 | 96981 | 0.06 | 6-Aminocaproic acid, N-allyloxycarbonyl-, octyl ester |
| 66 | 19.54 | 271993 | 0.16 | Tetrabutylammonium trifluoromethane sulfonate |
| 67 | 19.625 | 480302 | 0.28 | Tetrabutylammonium trifluoromethane sulfonate |
| 68 | 19.845 | 7719124 | 4.47 | 9-Octadecenoic acid, (E)-, TMS derivative |
| 69 | 20.059 | 898773 | 0.52 | Stearic acid, TMS derivative |
| 70 | 20.591 | 190653 | 0.11 | LINOLSAEURE, TRIMETHYLSILYLESTER |
| 71 | 21.145 | 603888 | 0.35 | 10-12-Pentacosadiynoic acid |
| 72 | 21.536 | 125796 | 0.07 | 2-Oleoylglycerol, 2TMS derivative |
| 73 | 22.071 | 272296 | 0.16 | cis-9-Hexadecenal |
| 74 | 22.195 | 295893 | 0.17 | 7-Oxooctanoic acid, TMS derivative |
| 75 | 22.403 | 225224 | 0.13 | Hexadecanoic acid, 2-hydroxy-1-(hydroxymethyl)ethyl este |
| 76 | 22.584 | 199041 | 0.12 | 2-Palmitoylglycerol, 2TMS derivative |
| 77 | 22.633 | 125018 | 0.07 | 1-Decanol, 8-[(trimethylsilyl)oxy]-, trifluoroacetate |
| 78 | 22.704 | 1450914 | 0.84 | Hexadecanoic acid, 4-[(trimethylsilyl)oxy]butyl ester |
| 79 | 22.844 | 722489 | 0.42 | 1-Monopalmitin, 2TMS derivative |
| 80 | 23.839 | 320054 | 0.19 | 2-Monostearin, 2TMS derivative |
| 81 | 23.902 | 193658 | 0.11 | NAPHTHALENE, 1,2,3,5,6,7,8,8A-OCTAHYDRO-1,8A- |
| 82 | 24.017 | 1017385 | 0.59 | 9,12-OCTADECADIENOIC ACID (Z,Z)-, 2-[(TRIMETHY |
| 83 | 24.189 | 639263 | 0.37 | OCTADECANOIC ACID, TRIMETHYLSILYL ESTER |
| 84 | 24.27 | 259443 | 0.15 | OCTADECANOIC ACID, 2,3-BIS[(TRIMETHYLSILYL) |
| 85 | 24.408 | 889171 | 0.51 | 9-OCTADECENAMIDE |
| 86 | 26.566 | 252890 | 0.15 | 1-OXA-3-ISOPROPYL-5-TRIMETHYLSILOXY-CYCLO |
| 87 | 27.875 | 873905 | 0.51 | Adipic acid, eicosyl 2-methylpent-3-yl ester |
| 88 | 30.05 | 623583 | 0.36 | HEXADECANOIC ACID, 2-[(TRIMETHYLSILYL)OXY] |
| 89 | 30.533 | 15138537 | 8.76 | Methyl 2-hydroxytetracosanoate, TMS derivative |
| 90 | 33.89 | 12270361 | 7.1 | 2,3-BIS[(TRIMETHYLSILYL)OXY]PROPYL 9-OCTADE |
| 91 | 39.552 | 17425120 | 10.08 | Methyl 2-hydroxytetracosanoate, TMS derivative |
| 92 | 42.83 | 10127888 | 5.86 | 2,3-BIS[(TRIMETHYLSILYL)OXY]PROPYL 9-OCTADE |
|  |  | 1.73E+08 | 100 |  |
